# Interplay Between Fibroblast Growth Factor-19, Beta-Klotho, and Receptors Impacts Cardiovascular Risk in Chronic Kidney Disease

**DOI:** 10.3390/jcm15031005

**Published:** 2026-01-27

**Authors:** Laura González-Rodríguez, Manuel Martí-Antonio, Virginia Díaz-Acevedo, Sonia Mota-Zamorano, Celia Chicharro, Bárbara Cancho, Raquel Gil-Lozano, Zoraida Verde, Fernando Bandrés, Nicolás R. Robles, Guillermo Gervasini

**Affiliations:** 1 Department of Medical and Surgical Therapeutics, Medical School, Universidad de Extremadura, 06006 Badajoz, Spain; lgonzalezn@alumnos.unex.es (L.G.-R.); vdiazace@alumnos.unex.es (V.D.-A.); ragill@alumnos.unex.es (R.G.-L.); 2 RICORS2040 Renal Research Network, 28029 Madrid, Spain; nrrobles@unex.es; 3 Vascular and Renal Translational Research Group, Institute for Biomedical Research in Lleida, 25198 Lleida, Spain; mmartia@irblleida.cat; 4 Department of Medical and Surgical Therapeutics, Plasencia University Center, Universidad de Extremadura, 10600 Plasencia, Spain; soniamz@unex.es; 5 Department of Biochemistry, Molecular Biology and Physiology, Universidad de Valladolid, 42004 Soria, Spain; celia.chicharro@uva.es (C.C.); zoraida.verde@uva.es (Z.V.); 6 GIR—Pharmacogenetics, Cancer Genetics, Genetic Polymorphisms and Pharmacoepidemiology, University of Valladolid, 47005 Valladolid, Spain; 7 Service of Nephrology, Badajoz University Hospital, 06006 Badajoz, Spain; barbara.cancho@salud-juntaex.es; 8 Biopathology-Toxicology Laboratory, Department of Legal Medicine, Psychiatry and Pathology, Faculty of Medicine, University Complutense of Madrid, 28040 Madrid, Spain; fbandres@med.ucm.es; 9 Institute of Molecular Pathology Biomarkers, Universidad de Extremadura, 06006 Badajoz, Spain

**Keywords:** chronic kidney disease, cardiovascular risk, FGF19, β-Klotho, risk prediction

## Abstract

**Background:** Chronic kidney disease (CKD) markedly increases the risk of cardiovascular events (CVE), yet conventional biomarkers often fail to capture this excess risk. We evaluated whether circulating levels and genetic variability within the FGF19/β-Klotho/FGFR axis contribute to CV risk stratification in CKD. **Methods:** In 579 CKD patients, plasma FGF19 and β-Klotho concentrations were quantified, and 64 genetic variants across FGF19, KLB, FGFR1, and FGFR4 genes were analyzed. **Results:** Cluster analysis identified three distinct biomarker profiles, with one cluster—characterized by low/intermediate FGF19 and markedly elevated β-Klotho—showing significantly reduced CV event-free survival. After adjustment for clinical covariates, this cluster was independently associated with higher CV risk [HR = 2.97 (1.12–7.92), *p* = 0.029]. Two genetic variants also showed independent associations: FGFR1 rs2288696 (protective) [HR = 0.51 (0.27–0.95), *p* = 0.029] and KLB rs2687971 (risk-increasing) [HR = 2.03 (0.97–4.27), *p* = 0.046]. A combined CV risk model incorporating biomarker clusters, relevant SNPs, and traditional risk factors achieved good discriminative ability (C-index = 0.80), with the FGF19/β-Klotho cluster showing predictive importance comparable to diabetes and previous CV history. **Conclusions:** These results indicate that integrating FGF19-Klotho biomarkers with genetic information may improve CV risk prediction in CKD.

## 1. Introduction

Chronic kidney disease (CKD) is becoming increasingly prevalent worldwide [1]. These patients, even in moderate stages of the disease, have a significantly increased risk of experiencing cardiovascular events (CVE) compared to the general population, despite adherence to evidence-based treatment guidelines [2,3]. In addition, because CKD progresses slowly and often without clear symptoms, early detection is crucial. Both lifestyle modifications and targeted drug therapies can help improve clinical outcomes in affected patients [4]. Consequently, there is a pressing need to identify new biomarkers of the disease, as traditional CKD markers only become evident when the disease is well advanced [5].

One of the most promising candidates in this regard is the endocrine fibroblast growth factor (FGF)-Klotho system. FGFs constitute a large family of signaling molecules that play pivotal roles in a wide array of cellular processes. Among these, FGF19, FGF21, and FGF23 operate as circulating hormones, regulating critical metabolic processes in distant tissues [6]. Interestingly, this FGF-Klotho system has been suggested to be associated with the risk and clinical course of CKD [7,8]. However, whilst FGF21 and, in particular, FGF23 have been extensively studied, significantly less data are available regarding the cardiorenal implications of FGF19.

**Figure 1:**
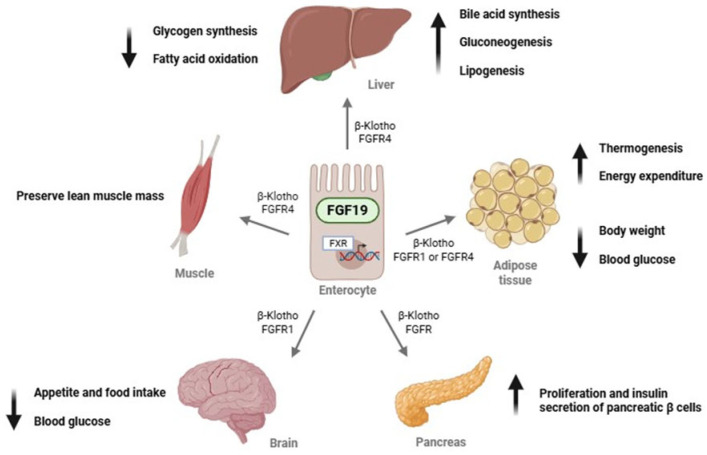
Endocrine actions of FGF19 in different target organs. Abbreviations: FGF19, fibroblast growth factor 19; FGFR1, fibroblast growth factor receptor 1; FGFR4, fibroblast growth factor receptor 4; FXR, farnesoid X receptor; β-Klotho, beta-Klotho protein.

The primary physiological role of FGF19 is the regulation of bile acid synthesis and subsequent cholesterol homeostasis [9], as well as modulation of insulin secretion and glycogen metabolism [10]. FGF19 requires β-Klotho to signal through two main receptors: FGFR1, which is primarily expressed in adipose tissue, and FGFR4, which is predominantly found in the liver [11,12]. Figure 1 shows the main biological functions of FGF19.

There are data linking FGF19 to conditions that are common in CKD patients, such as diabetes, dyslipidemia, or CV dysfunction [13,14,15,16]. However, only a few studies, with very limited sample sizes, have examined FGF19 levels in CKD. Marchelek-Mysliwiec et al. reported in two consecutive studies that FGF19 concentrations were elevated in patients with stages 2–4 [17,18], whilst Reiche et al. made the same observation for subjects with end-stage kidney disease [19]. Conversely, a Japanese study including 73 subjects did not find a correlation between renal function and FGF19 levels, although it was carried out in the general population [13]. Additionally, to our knowledge, no clinical studies have evaluated the impact of genetic variants within these genes, namely *KLB* (encoding β-Klotho), *FGF19*, *FGFR1,* and *FGFR4*, which hold the potential to influence circulating FGF19/β-Klotho levels and/or their biological activity.

This study aims to investigate the relevance of the FGF19/β-Klotho/FGFR system in CKD by evaluating the impact of both its circulating levels and genetic variants on these patients. Specifically, we will focus on how these genetic and non-genetic markers may be linked to the CV complications that frequently occur in patients with renal disease.

## 2. Patients and Methods

This was a cohort study, with a baseline biomarker/genetic assessment and prospective follow-up for incident CVE. A total of 599 Spanish Caucasian CKD patients in different stages of the disease were recruited between 2017 and 2023 and followed up during routine nephrology consultations at the Badajoz University Hospital (Spain). Twenty patients were lost to follow-up. Out of the final 579 patients, 174 (30.1%) had CKD1–2, 89 (15.4%) were in stage 3, and 316 (54.6%) were in stage 4/5. Inclusion criteria were having a diagnosis of CKD and being over 18 years of age. Exclusion criteria included breastfeeding and pregnancy, as well as the presence of an active infection and cancer. Patients who had undergone transplantation or had acute kidney injury were also excluded from this study. All participants gave written informed consent to take part in this study, which received approval from the Ethics Committee of Badajoz University Hospital and was conducted in compliance with the Declaration of Helsinki and its subsequent amendments.

### 2.1. Clinical Variables

This study was initiated with the collection of blood samples and the thorough review of clinical records to establish significant background and status of the patient. From that moment on, medical records of enrolled patients were systematically examined to collect information on kidney function and CV outcomes occurring during follow-up [median of 34.9 (28.4–60.0) months]. Participants were monitored through their regular visits to the nephrology service of the collaborating hospital.

Diagnostic and prognostic stratification of patients was made using the KDIGO classification. Estimation of renal function was carried out by creatinine clearance (mL/min), glomerular filtration rate (GFR) estimated by the CKD-EPI formula with creatinine. Proteinuria was defined as urinary protein excretion exceeding 500 mg over 24 h (or albuminuria greater than 300 mg/24 h).

CV outcomes registered included stroke, acute myocardial infarction, acute coronary syndrome, coronary revascularization procedures (angioplasty or bypass surgery), peripheral arterial disease with lower-limb ischemia, documented angina with positive stress testing, coronary catheterization, sudden cardiac death, and death due to CV causes.

### 2.2. Determination of Biomarkers Circulating Levels

Blood samples were obtained on the day of the hospital visit, with prompt separation of plasma, which was stored at −80 C until analysis. FGF19 concentrations were measured using the ELLA™ system (Bio-Techne, Minneapolis, MN, USA), an automated microfluidic ELISA platform requiring minimal sample volume. Cartridges with 32 wells were used, allowing the simultaneous analysis of various analytes. Diluted plasma (50 μL) was mixed with an equal volume of sample diluent and dispensed into each well, after which 1 mL of wash buffer was added to the appropriate buffer inlets. The automated immunoassay was then run using Simple Plex Runner software (version 3.9.0.28), encompassing instrument initialization, microfluidic partitioning of samples, and incubation within glass nanoreactor (GNR) channels preloaded with immobilized capture antibodies, biotinylated detection antibodies, and a streptavidin–fluorophore conjugate. Fluorescence signals were generated by laser excitation and subsequently detected. Relative fluorescence units obtained from each GNR were converted into FGF concentrations through inverse fitting to a manufacturer-provided master calibration curve. As each microfluidic channel contains three GNRs, all measurements were performed in triplicate for each sample, and mean values were reported.

β-Klotho determinations were conducted with commercially available ELISA kits (Bio-Techne, Minneapolis, MN, USA), as described in detail elsewhere [20]. Quantification was performed spectrophotometrically using a Multiskan EX microplate reader (Thermo, Waltham, MA, USA). The coefficient of variability (%CV) was calculated using the following formula: %CV = standard deviation/mean. The limits for %CV were set at 5% and 10% for intra-assay and inter-assay measurements, respectively. All calculated %CV values were within these limits. Wavelengths were measured at 450 nm and 540 nm, and calibration curves were between 31.3 and 2000 pg/mL.

### 2.3. Genetic Analyses

The DNA of the participants was extracted from whole blood samples using a conventional phenol–chloroform purification protocol followed by ethanol precipitation. Four genes—*FGF19*, *KLB*, *FGFR1*, and *FGFR4*—were analyzed using two complementary strategies. First, tag single-nucleotide polymorphisms (tag-SNPs) representing the genetic variability across each gene locus were selected. To this end, all SNPs reported for individuals of European ancestry were retrieved from the International Genome Project database and imported into Haploview v. 4.2 software through conversion of Ensembl VCF files to PED format. Applying an r^2^ threshold of 0.8 and a minimum minor allele frequency of 0.05, the Haploview tagging algorithm identified 49 tag-SNPs by pairwise tagging, collectively capturing 100% of the known genetic variability in these four genes within the European population.

In addition, 15 variants with previously reported functional or clinical relevance were included in the analyses. Genotyping was carried out using a custom-designed panel targeting the selected variants on a QuantStudio™ 12K Flex Real-Time PCR System (Life Technologies, Carlsbad, CA, USA), employing TaqMan^®^ OpenArray technology. Each genotyping run included quality control samples, consisting of trio DNA samples obtained from the Coriell Institute Biorepository. All analyses were performed at the Centro Nacional de Genotipado–Instituto de Salud Carlos III (CeGen-ISCIII; Madrid, Spain, www.cegen.org). Supplementary Table S1 shows characteristics of the analyzed SNPs in our cohort.

### 2.4. Statistical Analysis

Categorical variables were presented as absolute counts and percentages, whereas continuous variables were described as medians and interquartile ranges (IQR, p25–p75). To evaluate the relationship between CKD stage and continuous variables, nonparametric Kruskal–Wallis tests were applied, given that clinical data did not follow a normal distribution. Associations between CKD stage and categorical variables were evaluated using the likelihood-ratio test (LRT). A multivariable multinomial logistic regression model was used to compare FGF19 and β-klotho concentrations across CKD stages. In addition, the associations between these biomarkers and eGFR were assessed using a multivariable linear regression model. Models were adjusted for covariates associated with CKD stage, as displayed in Table 1. To ensure consistency across regression approaches, all predictors were standardized prior to model fitting. Cox proportional hazards regression models were applied to examine the relationship between clinical variables and genetic variants with CV event-free survival, assuming a dominant model of inheritance, as it produced more balanced groups for comparison.

Across the entire cohort, a cluster analysis was conducted to classify patients based on their FGF19 and β-Klotho concentrations. The two variables were standardized prior to the analysis, and 17 patients were excluded as outliers according to Density-based Spatial Clustering of Applications with Noise (DB-SCAN) algorithm. The Euclidean partitioning around medoids (PAM) algorithm then identified three distinct clusters, which were visualized in a scatter plot. These clusters were subsequently analyzed in relation to CKD severity and displayed in a bar plot. Associations between clusters and CV event-free survival risk were assessed using Kaplan–Meier survival curves and Cox proportional hazards regression models adjusted for age, sex, BMI, hypertension, diabetes, smoking status, previous CV events, and CKD stage. These covariates were selected based on clinical relevance and/or results from univariate analyses. Results were expressed as hazard ratios (HR) with 95% confidence intervals (CI). The impact of SNP pair interactions in the participating genes on CV risk was evaluated using log likelihood-ratio tests and controlling for age, sex, BMI, hypertension, diabetes, smoking, and previous CV history. In the charts illustrating these analyses, the upper triangle of the matrix displays the *p*-values for the interaction log likelihood-ratio test, whilst the lower triangle presents the *p*-values from the likelihood-ratio test, which compares the additive likelihood of two variants to the best of the single-variant models. The diagonal line shows the *p*-values derived from the likelihood-ratio test for the unadjusted effect of each variant.

An Elastic-Net-regularized Cox proportional hazards model was fitted to estimate the risk of CVE. Predictors included concentration clusters, relevant SNP variants (rs10400230, rs2687971, rs2687963, rs17580578, and rs2288696), as well as adjustment covariates. All predictors were standardized before being entered into the model. A 10-fold cross-validation based on partial likelihood deviance was used to identify the regularization parameter (λ) that minimized the standard error. The Elastic-Net mixing parameter (α) was set to 0.5 to balance the LASSO and Ridge penalties, providing a compromise between variable selection and the retention of correlated predictors. Variable importance was assessed based on the absolute value of each coefficient in the model, scaled to 100%, and presented in a bar plot. Model discrimination was quantified using the concordance index (C-index) for right-censored data.

With 579 individuals included in this study, a CVE incidence of 9.0% (52 patients), and a two-sided alpha of 0.05, this study had a statistical power of 85.5% to detect a standardized effect size (Cohen’s d) of 0.45 between patients with and without CVE. Power calculations were performed using the Wilcoxon–Mann–Whitney test in G*Power (version 3.1.9.6, Kiel University, Kiel, Germany).

All statistical procedures were carried out using R v. 4.3.3 software and several dedicated packages. Kaplan–Meier curves were generated with survminer. Multinomial logistic regression model was performed with VGAM. Cox proportional hazards regression models were fitted with survival. Cluster analysis was conducted with cluster and dbscan. Scatter and bar plots were produced using ggplot2. SNP pair interactions were evaluated with SNPassoc. Elastic-Net-regularized Cox proportional hazards model was obtained with glmnet. A *p*-value < 0.05 was considered statistically significant.

During the preparation of this work, the authors used ChatGPT 5.1 in order to improve the readability and language of the manuscript. After using this tool, the authors reviewed and edited the content as needed and take full responsibility for the content of the publication.

## 3. Results

Out of the 579 CKD patients, 61.8% were men and 38.2% were women, a distribution that did not change significantly among stage groups (*p* = 0.133). Median (IQR) age of patients was 67 (57–76) years, with age increasing significantly with CKD severity (*p* < 0.001). Median eGFR in the CKD cohort was 25.0 (16.0–73.9) mL/min/1.73 m^2^. Regarding the incidence of CV risk factors, patients at CKD stage 1–2 showed fewer cases of hypertension, dyslipidemia, diabetes, and previous history of CV events (*p* < 0.05 in all cases for the difference between the three study groups). Table 1 shows these and other clinical and demographic characteristics of the participants. The most frequent cause of CKD in our cohort was diabetic nephropathy (20.4%), followed by nephroangiosclerosis (18.0%) and interstitial nephropathy (13.5%). The cause could not be determined in 14.2% of cases.

### 3.1. FGF19 and β-Klotho Concentrations in Chronic Kidney Disease

Median (IQR) of FGF19 and β-Klotho concentrations in the cohort were 210 (142–319) and 1083 (762–1552) pg/mL, respectively. Table 1 displays values of the two biomarkers in the three-stage groups. Taking as reference the CKD1–2 group, multinomial regression analyses showed that FGF19 was significantly increased in the patients with more severe CKD (*p* < 0.0001), while β-Klotho reached the highest values in the CKD 3 stage group (*p* = 0.003).

Analyses were also conducted considering eGFR values instead of CKD stages. A linear regression model was built, which showed that concentrations of β-Klotho (−2.903, *p* = 0.023), but especially FGF19 (−6.535, *p* < 0.0001), were inversely correlated with renal function. Supplementary Table S2 shows both the multinomial and linear models utilized.

### 3.2. Clusterization of FGF19 and β-Klotho Concentrations in Chronic Kidney Disease

**Figure 2:**
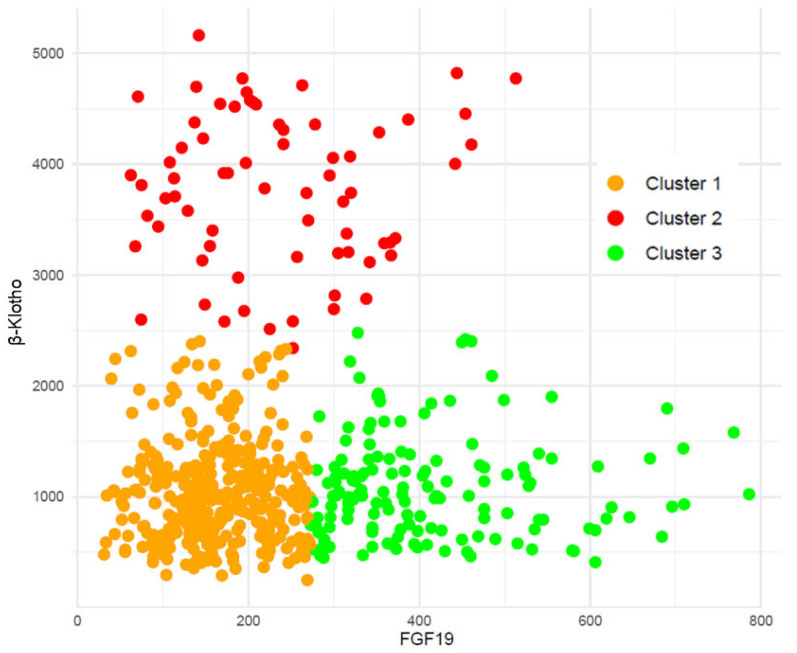
Cluster analysis grouped both β-Klotho and FGF19 plasma concentrations in the cohort of renal patients.

We then performed cluster analyses to group our patients according to these biomarkers’ concentrations. Figure 2 depicts the three clusters obtained. Median (IQR) values of FGF19 and β-Klotho for each cluster were, respectively, cluster 1: 161 (121–211) and 1001 (692–1286) pg/mL; cluster 2: 219 (147–311) and 3811 (3259–4358) pg/mL; and cluster 3: 379 (323–476) and 1007 (717–1324) pg/mL.

**Figure 3:**
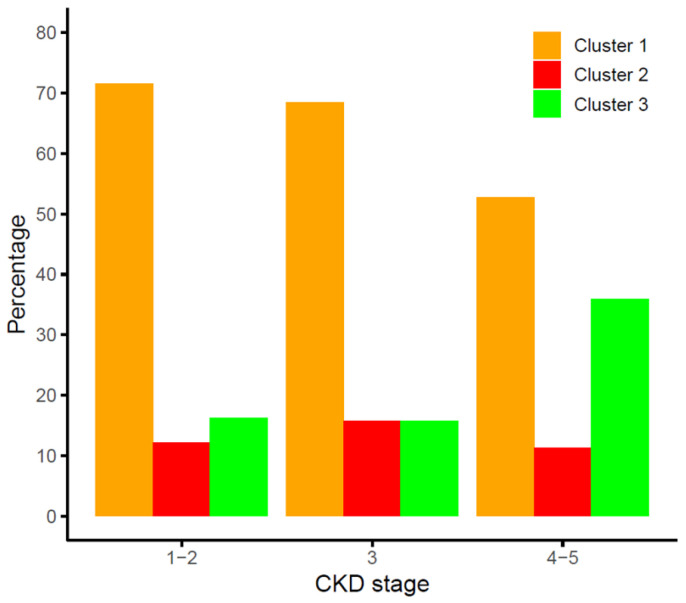
Distribution of the FGF19/β-Klotho cluster groups across stages of chronic kidney disease (CKD).

The distribution of the clusters among the three CKD stage groups studied is shown in Figure 3. The distributions of clusters 1 and 3 were significantly different across CKD stages (*p* < 0.0001), whilst the percentage of patients within the high-risk cluster 2, showing high β-Klotho and low–intermediate FGF19 concentrations, was similar for all CKD stages (*p* = 0.551).

### 3.3. Effect of Combined FGF19/β-Klotho Concentrations on Cardiovascular Risk in CKD Patients

**Figure 4:**
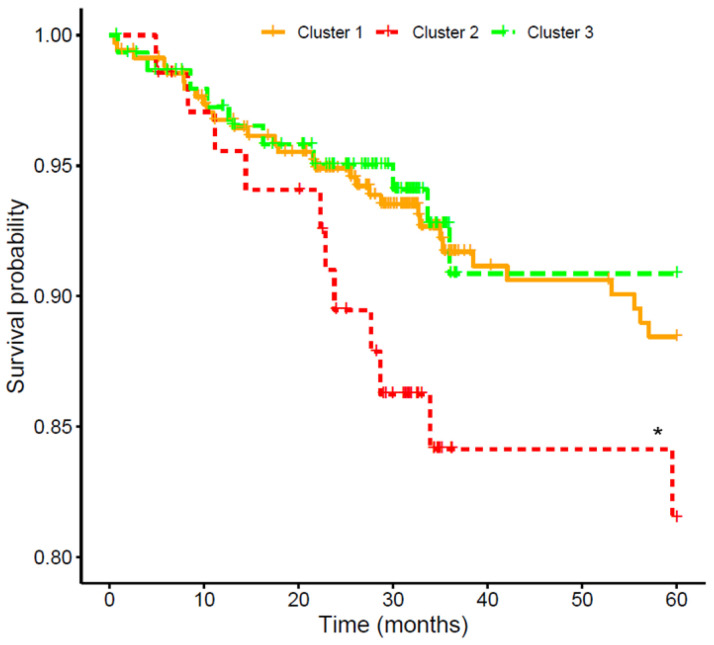
Kaplan–Meier curves for the association of FGF19/β-Klotho cluster groups with cardiovascular event-free survival in chronic kidney disease patients. * Cox-adjusted *p* < 0.05 vs. cluster 3.

To assess CV risk in the cohort, the 579 participants were followed for a median of 34.9 (28.4–60.0) months to register the incidence of CVE, which was experienced by 52 (9.0%) patients. Table 2 shows demographic and clinical characteristics of patients who did or did not suffer a CVE. As expected, subjects that suffered CVE were older (*p* < 0.001), had elevated concentrations of glucose (*p* = 0.001), cholesterol (total, HDL, and LDL, *p* < 0.01), and albuminuria (*p* < 0.001), along with a higher incidence of previous CVE (*p* < 0.001) and diabetes (*p* < 0.001). The association of the three FGF19/β-Klotho clusters with CV event-free survival is shown in Figure 4. Kaplan–Meier analysis revealed that patients within cluster 2 had a lower event-free survival (84.1%) than those in clusters 1 (91.0%) and 3 (93.3%). After adjusting a Cox regression model for age, sex, BMI, hypertension, diabetes, smoking, CKD stage, and previous CV history, cluster 2 presented a significantly higher CV risk compared to cluster 3 [HR = 2.97 (1.12–7.92), *p* = 0.029].

### 3.4. Association of Genetic Variants in the FGF19-Klotho System with Cardiovascular Risk

**Figure 5:**
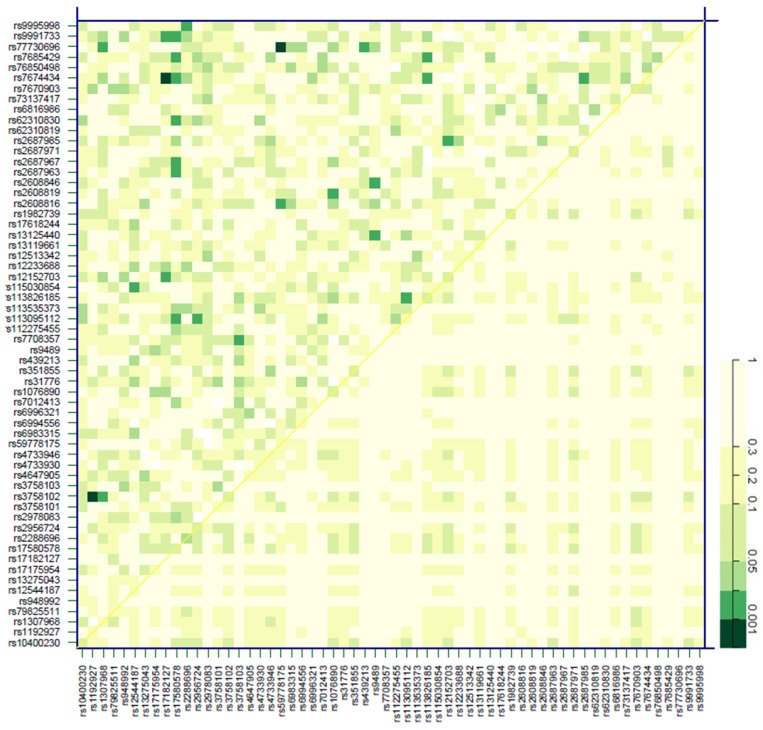
Effect of SNP pair interactions in the *FGF19*, *KLB*, *FGFR1,* and *FGFR4* genes on the cardiovascular risk of patients with chronic kidney disease. The diagonal line contains the *p*-values from the likelihood-ratio test for the crude effect of each SNP. The upper triangle in the matrix contains the *p*-values for the interaction (epistasis) log likelihood-ratio test. The lower triangle contains the *p*-values from the likelihood-ratio test comparing the two-SNP additive likelihood to the best of the single-SNP models. The colored bar denotes the *p*-values for the observed associations.

Next, we assessed the impact of variability in the participating genes (*FGF19*, *KLB*, *FGFR1,* and *FGFR4*) on the incidence of CVE in the cohort. Cox regression models adjusted by the aforementioned covariates revealed that rs2288696 in FGFR1 displayed a protective effect [HR = 0.51 (0.27–0.95), *p* < 0.029], whilst *KLB* rs2687971 increased CV risk [HR = 2.03 (0.97–4.27), *p* = 0.043] (Table 3). Figure 5 shows SNP pair interactions between genetic variability in the four assayed genes with regard to their effect on CV events. Three pairs, namely *FGF19* rs1192927-*FGFR1* rs3758102 (*p* < 0.001), *FGFR1* rs17182127-*KLB* rs7674434 (*p* < 0.001), and *FGFR1* rs59778175-*KLB* rs77730696 (*p* < 0.001), showed a marked impact on CV risk.

### 3.5. Combined Risk Model for Cardiovascular Risk in Chronic Kidney Disease

**Figure 6:**
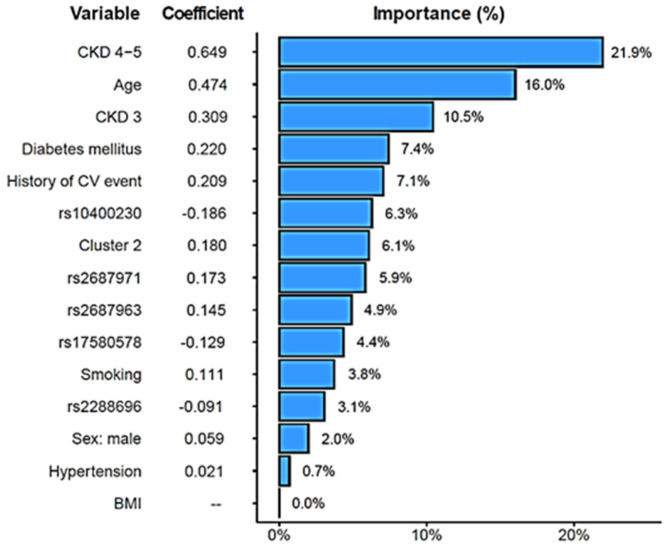
Elastic-Net-regularized Cox proportional hazard model to estimate the risk of cardiovascular events. Coefficients and relative importance of each variable in the model are shown.

A combined CV risk model for the CKD patients was created that included the FGF19/β-Klotho concentration cluster, relevant genetic variants [the two SNPs with significant CV associations, rs2288696, rs2687971, and three more variants with suggestive *p*-values (<0.1), namely, rs17580578, rs10400230, and rs2687963], as well as age, sex, BMI, hypertension, diabetes, smoking, CKD stage, and previous CV history, as described in previous analyses. The model achieved a C-index of 0.80. Figure 6 shows that, as expected, CKD severity and age carried the largest weight in the model (importance ranging from 10.5 to 21.9%). Remarkably, the risk cluster showed a coefficient of 0.180 with a 6.1% importance in the model, figures that were similar to those shown by diabetes (0.220, 7.4%) or previous CV history (0.209, 7.1%) and far superior to other risk factors such as smoking (0.111, 3.8%), hypertension (0.021, 0.7%), or BMI (0%, left out of the model). The rs10400230 SNP was the variant displaying the highest coefficient (−0.186, 6.3% importance).

## 4. Discussion

CV diseases account for 40–50% of deaths in CKD patients [21,22]. In the present study, we have evaluated the putative role of FGF19 and its necessary cofactor β-Klotho and show that both their plasma levels and the variability in their encoding genes can modify the incidence of CV complications in this high-risk population.

It has recently been hypothesized that alterations in circulating concentrations of FGF19 in CKD patients may also affect CV risk, due to its role in regulating crucial metabolic pathways [16]. Indeed, our results show that patients with both low/intermediate levels of FGF19 and high levels of β-Klotho (cluster 2) were at significantly elevated CV risk, as compared with those with high FGF19 and low β-Klotho. It should be noted that patients within this risk cluster were uniformly distributed across all CKD stages, and therefore, disease severity was not a confounding factor in their observed increased CV risk. To date, there are no studies that have analyzed the association of combined FGF19/β-Klotho concentrations with CV risk, and only a few have examined their individual impact, with none of them in the CKD setting. Regarding FGF19, and in line with the data presented herein, low FGF19 plasma levels have recently been associated with diminished cardiac function [23] and worse prognosis in patients with acute ischemic stroke [24]. In contrast, Hu et al. showed that elevated FGF19 serum levels correlated with atherosclerosis severity in men with type 2 diabetes [25]. However, the reported mean values in the latter study, even though they were considered elevated, were in fact lower than the mean concentration in our patients at high CV risk (171.2 pg/mL vs. 219.0 pg/mL). These findings underscore the complexity of FGF19-relatedCV risk and the potential influence of the specific disease context, such as the presence or absence of diabetes or renal injury, as is the case in this study. In this regard, the severity of the renal disease correlated positively with plasma FGF19 levels in our patients, which is in agreement with three previous studies in CKD [17,18,19]. The large number of patients included in our cohort supports the hypothesis that there is indeed an inverse association between FGF19 concentrations and, to a lesser extent, β-Klotho and eGFR in CKD. Furthermore, studies in control subjects report FGF19 levels that are significantly lower than those observed in renal patients [26,27]. It is possible that, similar to FGF21, dysregulated concentrations of FGF19 in CKD patients result from metabolic alterations, inflammation, and probably even nutritional factors [17].

The other member of the combined biomarker was β-Klotho, which has been proposed as a promising drug target for metabolic and CV disease [28]. However, unlike its alpha analogue [29], there is no information on the putative impact of its circulating concentrations, particularly in the CKD setting. Our findings show that subjects in cluster 2, with elevated plasma levels of β-Klotho, had a higher incidence of CVE. A hypothesis could be that, since these at-risk patients also had low FGF19 concentrations, the excess of β-Klotho would be free to interact and activate FGF21, hence triggering a signal that could end up increasing CV risk [30]. Indeed, high blood levels of FGF21 have been shown to be an independent risk factor for heart failure [31,32]. In agreement with this hypothesis that remarks the importance of β-Klotho concentrations, neither cluster 1 nor cluster 3—both with low Klotho levels—showed an increased CV risk. It should be noted that cluster 1, with lower FGF19 values, displayed a slightly poorer survival, although the difference with cluster 3 was not significant. Nevertheless, whether the CV impact is driven by the β-Klotho interplay with FGFs or by independent mechanisms warrants further investigation.

A second aspect of the study dealt with the putative effect of genetic variability in the genes coding for all the players in this system: FGF19, β-Klotho, FGFR1, and FGFR4. Our findings revealed that rs2288696 in *FGFR1* exerted a protective effect against CV risk. This is an intronic SNP that has not been evaluated before in the cardiorenal context, although interestingly enough, it has also been associated with a protective effect in ovarian cancer [33]. FGFRs are essential mediators of the biological effects of FGFs, and hence, it is plausible that genetic variations in FGFRs may affect CV function. For instance, FGFR1 signaling has been implicated in the development of kidney injury associated with hypertension [34]. Furthermore, a very recent report points to *FGFR1* as a novel locus and therapeutic target for cardiometabolic health [35]. The other variant with a significant impact on CV risk was rs2687971 in the *KLB* gene, which codes for β-Klotho and whose locus variability has recently been implicated in cardiometabolic complications [36] and the incidence of CVE [37]. *KLB* rs2687971 is located in the 3′-UTR region of the gene and could therefore be involved in mRNA stability or miRNA binding [38], although no clinical repercussions of this variant have been reported as yet. In any case, although the *p*-value for the association was significant, the lower limit of the confidence interval was 0.97, and therefore, caution should be exerted when considering this result. In addition, our findings also showed that variants present in the locus of different genes that need to work together to trigger biological functions (*FGF19*/*FGFR1*/*KLB*) may also interact to significantly affect these functions, in this case altering CV risk. In this regard, we have previously reported that interactions between SNPs in glucose homeostasis genes can also impact the susceptibility to CVE in patients with diabetic nephropathy [39].

Finally, we included both the FGF19/β-Klotho cluster and the relevant genetic variants in a CV risk model for CKD patients that showed a C-index of 0.80, which is considered good/excellent discriminative ability in other CV clinical studies [40,41]. The cluster and some of the variants carried a similar weight in the model as that shown by diabetes or previous CV history while being superior to smoking, sex, hypertension, or BMI. Traditional models often fall short in accurately predicting CV events in CKD. We have previously shown how CV risk models in CKD can be significantly enhanced by the addition of novel biomarkers—e.g., endothelin [42], and genetic variants, e.g., those in the prostaglandin E2 pathway [43]—or even nutritional information [44]. Taken together, these previous findings and our current data suggest that conventional markers still have considerable limitations and that risk models can and must be efficiently tuned up. There is a pressing need for novel CV biomarkers in renal disease, especially considering that CKD is projected to rank among the five leading causes of death globally by 2040, largely driven by its CV complications [45].

This study has several strengths and limitations. To our knowledge, it is the first to examine the FGF19/β-Klotho system in renal patients to assess CV risk. In addition, the parallel genetic analysis enabled the identification of relevant variants that can enhance the performance of CV risk models. Among the limitations, first, a control group was not available in this study; second, we did not measure the expression of FGF19/β-Klotho in the organs of interest, whose relationship with circulating levels would be most informative. Additionally, the genetic study design included the analysis of tag-SNPs, i.e., variants that represent genetic variability in a certain region but that usually lack a known functional impact, being mostly intronic. This means that, while we were able to capture the whole variability of each gene, connecting the observed genotype–phenotype association to a particular biochemical consequence of the SNP is challenging. Finally, the reported CV risk model should obviously undergo external validation before considering clinical use.

## 5. Conclusions

In summary, we have demonstrated how the FGF19/β-Klotho system plays a relevant role in CKD. Specifically, a concentration cluster integrating FGF19 and β-klotho concentrations could function as a biomarker of CV risk in the renal setting. Its integration into a CV risk prediction model, alongside pertinent genetic markers and established risk factors, has the potential to enhance the prevention and clinical management of CV complications in individuals with CKD.

## Figures and Tables

**Table 1:** Characteristics of the studied patients with chronic kidney disease stratified by disease stage.

	CKD 1–2 (*n* = 174)	CKD 3 (*n* = 89)	CKD 4–5 (*n* = 316)	* *p*
Males (%)	97 (55.7%)	59 (66.3%)	202 (63.9%)	0.133
Age (Years)	58.0 (49.0–67.0)	66.0 (60.0–75.0)	71.0 (60.0–79.3)	<0.0001
Weight (Kg)	81.0 (66.3–91.4)	79.7 (73.0–90.5)	78.1 (67.1–89.1)	0.108
BMI	28.3 (25.1–31.1)	29.5 (26.8–32.4)	28.8 (25.5–32.7)	0.099
Glucose (mg/dL)	100.5 (93.0–112.0)	111.0 (97.0–145.0)	101.0 (90.0–119.3)	<0.0001
Total cholesterol (mg/dL)	173.0 (154.3–196.8)	158.0 (138.0–199.0)	144.0 (122.8–171.3)	<0.0001
HDL cholesterol (mg/dL)	54.0 (44.0–64.0)	46.0 (37.0–54.0)	45.0 (37.0–57.0)	<0.0001
LDL cholesterol (mg/dL)	96.0 (79.0–114.0)	83.0 (62.0–109.5)	68.7 (51.0–92.0)	<0.0001
Total calcium (mg/dL)	9.4 (9.3–9.7)	9.6 (9.2–9.8)	9.3 (8.9–9.6)	<0.0001
Potassium (mEq/L)	4.3 (4.1–4.6)	4.7 (4.4–5.1)	5.0 (4.5–5.3)	<0.0001
Sodium (mEq/L)	141.0 (140.0–143.0)	142.0 (140.0–143.0)	141.0 (139.0–142.0)	0.0001
ACR (mg/g) in urine 24 h	8.4 (4.2–31.3)	97.7 (12.4–281.9)	410.0 (139.4–1180.0)	<0.0001
eGFR (mL/min/1.73 m^2^)	98.9 (83.3–106.8)	40.9 (33.7–49.0)	16.4 (13.0–20.0)	<0.0001
Systolic blood pressure (mmHg)	132.0 (123.0–147.0)	147.0 (129.5–164.0)	144.0 (127.0–163.3)	<0.0001
Diastolic blood pressure (mmHg)	80.0 (74.0–89.0)	80.0 (67.5–87.5)	74.0 (66.0–85.0)	<0.0001
Pulse pressure (mmHg)	51.0 (43.0–64.0)	67.0 (53.5–83.0)	69.0 (51.0–86.3)	<0.0001
Hypertension (%)				0.042
No	40 (23.0%)	16 (18.0%)	44 (13.9%)	
Yes	134 (77.0%)	73 (82.0%)	272 (86.1%)	
Diabetes (%)				<0.0001
No	143 (82.2%)	40 (44.9%)	167 (52.8%)	
Yes	31 (17.8%)	49 (55.1%)	149 (47.2%)	
Smoking (%)				0.304
Non-smoker	84 (48.8%)	33 (38.8%)	140 (44.4%)	
Ever smoker	88 (51.2%)	52 (61.2%)	175 (55.6%)	
Hyperlipidemia (%)				<0.0001
No	120 (69.0%)	40 (44.9%)	90 (28.7%)	
Yes	54 (31.0%)	49 (55.1%)	224 (71.3%)	
β-Klotho (pg/mL)	852 (605–1344)	1345 (1.156–2065)	1079 (798–1482)	<0.0001
FGF19 (pg/mL)	168 (122–237)	165 (114–236)	260 (176–359)	<0.0001

ACR, albumin to creatinine ratio; BMI, body mass index; CKD, chronic kidney disease; eGFR, estimated glomerular filtration rate. * *p*-value for the difference between the three groups.

**Table 2:** Demographic and clinical variables of participants according to the presence or absence of cardiovascular complications during follow-up.

	No CVE (*n* = 527)	CVE (*n* = 52)	*p*-Value
Males (%)	322 (61.1%)	36 (69.2%)	0.214
Age (Years)	66 (56–75)	72 (66–78)	<0.0001
Weight (Kg)	79 (68–90)	79 (69–90)	0.417
BMI	28.7 (25.5–32.5)	28.9 (25.6–31.8)	0.751
Glucose (mg/dL)	101.0 (91.0–117.0)	123.0 (96.5–150.5)	0.001
Total cholesterol (mg/dL)	158 (136–184)	129 (117–175)	<0.0001
HDL cholesterol (mg/dL)	48 (39–60)	43 (36–52)	0.001
LDL cholesterol (mg/dL)	82 (61–104)	60 (51–93)	0.005
Total calcium (mg/dL)	9.4 (9.1–9.7)	9.3 (8.9–9.6)	0.116
Potassium (mEq/L)	4.7 (4.3–5.1)	4.8 (4.4–5.3)	0.613
Sodium (mEq/L)	141 (139–143)	141 (139–142)	0.990
ACR (mg/g) in urine 24 h	156.5 (14.2–594.1)	377.9 (73.3–1056.7)	<0.0001
Troponin	33.3 (21.5–51.6)	49.3 (34.8–68.5)	0.045
NT_proBNP	796 (307–2092)	2923 (698–6538)	0.007
eGFR (ml/min/1.73 m^2^)	26.0 (16.0–81.5)	20.0 (15.0–42.3)	<0.0001
Systolic blood pressure (mmHg)	140 (125–159)	149 (132–167)	0.082
Diastolic blood pressure (mmHg)	77 (69–87)	74 (66–84)	0.025
Pulse pressure (mmHg)	61 (47–78)	73 (60–86)	0.001
Hypertension (%)			0.128
No	94 (17.8%)	6 (11.5%)	
Yes	433 (82.2%)	46 (88.5%)	
History of CV event (%)			<0.0001
No	395 (75.4%)	28 (54.9%)	
Yes	129 (24.6%)	23 (45.1%)	
Diabetes (%)			<0.0001
No	330 (62.6%)	20 (38.5%)	
Yes	197 (37.4%)	32 (61.5%)	
CKD stages			<0.0001
CKD 1–2	170 (32.3%)	4 (7.7%)	
CKD 3	75 (14.2%)	14 (26.9%)	
CKD 4–5	282 (53.5%)	34 (65.4%)	
Smoking (%)			0.308
Non-smoker	238 (45.6%)	19 (38.0%)	
Ever smoker	284 (54.4%)	31 (62.0%)	
Hyperlipidemia (%)			0.009
No	234 (44.6%)	16 (30.8%)	
Yes	291 (55.4%)	36 (69.2%)	
Patients within each cluster			
Cluster 1	312 (61.2%)	31 (59.6%)	0.565
Cluster 2	58 (11.4%)	11 (21.2%)	0.062
Cluster 3	140 (27.5%)	10 (19.2%)	0.422

BMI, body mass index; ACR, albumin to creatinine ratio; CV, cardiovascular; CKD, chronic kidney disease.

**Table 3:** Significant associations of genetic variants with cardiovascular event-free survival. HR, hazard ratio.

Genetic Variant	Genotype	No CVE	CVE	HR (95% CI)	*p*-Value
*FGFR1* rs2288696	G/G	87.6%	12.4%	Reference	
	A/G, A/A	91.5%	8.5%	0.51 (0.27,0.95)	0.029
*KLB* rs2687971	C/C	91.9%	8.1%	Reference	
	CG, GG	88.1%	11.9%	2.03 (0.97,4.27)	0.046

CVE, cardiovascular events; HR, hazard ratio; CI, confidence intervals.

## Data Availability

Source data for this study have been uploaded to the Figshare repository and are openly available at https://figshare.com/articles/dataset/FGF_betaKlotho_in_CKD/30720536?file=59866880 (accessed on 1 December 2025) with DOI 10.6084/m9.figshare.30720536.

## Supplementary Materials

The following supporting information can be downloaded at https://www.mdpi.com/article/10.3390/jcm15031005/s1. Table S1: Characteristics of the genetic variants analyzed. Table S2: Multinomial and linear regression models evaluating the association of FGF19 and β-Klotho concentrations with CKD stages and renal function estimated by glomerular filtration rate (eGFR).

## Abbreviations

The following abbreviations are used in this manuscript:

CKD	Chronic kidney disease
CVE	Cardiovascular event
SNP	Single-nucleotide polymorphism
eGFR	Estimated glomerular filtration rate
HR	Hazard ratio
ACR	Albumin-to-creatinine ratio
BMI	Body mass index
