# Supplementary material for: Interplay Between Fibroblast Growth Factor-19, Beta-Klotho, and Receptors Impacts Cardiovascular Risk in Chronic Kidney Disease
Source: J Clin Med. 2026 Jan 27;15(3):1005. doi: 10.3390/jcm15031005 (PMC12897728; doi:10.3390/jcm15031005)
Supplement: Supplementary file 1 [file jcm-15-01005-s001.zip › jcm-4076589-Supplementary.pdf]

Supplementary Table S1. Characteristics of the genetic variants analyzed

Gene	Variant	Position	Reference allele	Alternate allele	MAF	Location / Consequence
FGF19	rs10400230	11:69700202	T	C	0.051	Intronic
FGF19	rs1192927	11:69699889	A	C	0.328	Intronic
FGF19	rs1307968	11:69700655	A	G	0.129	Intronic
FGF19	rs79825511	11:69700928	G	A	0.123	Intronic
FGF19	rs948992	11:69698570	A	G	0.310	3' UTR
FGFR1	rs12544187	8:38466908	C	A	0.432	Intronic
FGFR1	rs13275043	8:38460819	G	C	0.488	Intronic
FGFR1	rs17175954	8:38422351	C	T	0.050	Intronic
FGFR1	rs17182127	8:38466823	G	A	0.131	Intronic
FGFR1	rs17580578	8:38450649	T	C	0.117	Intronic
FGFR1	rs2288696	8:38428707	G	A	0.217	Intronic
FGFR1	rs2956724	8:38434748	C	A	0.059	Intronic
FGFR1	rs2978083	8:38431385	C	T	0.023	Intronic
FGFR1	rs3758101	8:38459958	C	A	0.216	Intronic
FGFR1	rs3758102	8:38459331	C	T	0.226	Intronic
FGFR1	rs3758103	8:38457883	T	C	0.131	Intronic
FGFR1	rs4647905	8:38415024	C	G	0.192	Intronic
FGFR1	rs4733930	8:38453483	C	T	0.422	Intronic
FGFR1	rs4733946	8:38461831	G	T	0.085	Intronic
FGFR1	rs59778175	8:38447776	C	T	0.222	Intronic
FGFR1	rs6983315	8:38441901	G	A	0.423	Intronic
FGFR1	rs6994556	8:38467098	A	G	0.484	Intronic
FGFR1	rs6996321	8:38464828	A	G	0.402	Intronic
FGFR1	rs7012413	8:38459880	C	T	0.312	Intronic
FGFR4	rs1076890	5:177098070	G	A	0.058	3' UTR
FGFR4	rs31776	5:177096596	G	A	0.322	Splicing
FGFR4	rs351855	5:177093242	G	A	0.301	Missense (Gly388Arg)
FGFR4	rs439213	5:177091314	C	A	0.289	Intronic
FGFR4	rs9489	5:177098028	G	C	0.134	3' UTR
FGFR4	rs7708357	5:177104771	G	A	0.418	Intergenic
KLB	rs112275455	4:39418727	C	*T*	0.071	Intronic
KLB	rs113095112	4:39445546	C	*T*	0.196	Intronic
KLB	rs113535373	4:39425988	C	*G*	0.129	Intronic
KLB	rs113826185	4:39442199	G	*A*	0.069	Intronic
KLB	rs115030854	4:39442416	T	*C*	0.050	Intronic
KLB	rs12152703	4:39420199	G	*T*	0.241	Intronic
KLB	rs12233688	4:39411290	C	*T*	0.345	Intronic
KLB	rs12507413	4:39439870	G	*A*	0.455	Intronic
KLB	rs12513342	4:39447786	T	*C*	0.296	Intronic
KLB	rs13119661	4:39450856	C	*A*	0.130	3' UTR
KLB	rs13125440	4:39420704	A	*G*	0.371	Intronic

KLB	rs17618244	4:39446909	G	*A*	0.224	Missense (Arg728Gln)
KLB	rs1982739	4:39410419	A	*C*	0.151	Intronic
KLB	rs2608816	4:39425189	T	*C*	0.100	Intronic
KLB	rs2608817	4:39425581	T	*C*	0.402	Intronic
KLB	rs2608819	4:39429811	C	*T*	0.135	Intronic
KLB	rs2608846	4:39423059	G	*T*	0.272	Intronic
KLB	rs2687962	4:39439541	C	*T*	0.139	Intronic
KLB	rs2687963	4:39436529	A	*G*	0.312	Intronic
KLB	rs2687967	4:39448204	A	*G*	0.304	Intronic
KLB	rs2687971	4:39451324	C	*G*	0.478	3' UTR
KLB	rs2687985	4:39427593	T	*G*	0.334	Intronic
KLB	rs62310819	4:39409787	C	*T*	0.235	Intronic
KLB	rs62310830	4:39432197	C	*G*	0.191	Intronic
KLB	rs6531716	4:39424705	C	*T*	0.450	Intronic
KLB	rs6816986	4:39443510	C	*A*	0.097	Intronic
KLB	rs73137417	4:39427873	T	*C*	0.055	Intronic
KLB	rs7670903	4:39441340	G	*A*	0.419	Intronic
KLB	rs7674434	4:39417789	T	*G*	0.291	Intronic
KLB	rs76850498	4:39419205	T	*C*	0.055	Intronic
KLB	rs7685429	4:39446922	G	*C*	0.228	Synonymus (Pro732Pro)
KLB	rs77730696	4:39433587	C	*T*	0.082	Intronic
KLB	rs9991733	4:39419374	A	*G*	0.288	Intronic
KLB	rs9995998	4:39450670	C	*T*	0.171	3' UTR

MAF, minor allele frequency in the cohort

Supplementary Table S2. Multinomial and linear regression models evaluating the association of FGF19 and β-Klotho concentrations with CKD stages and renal function estimated by glomerular filtration rate (eGFR)

	**Multinomial regression**				**Linear regression**	
	**CKD 3 vs 1-2**		**CKD 4-5 vs 1-2**		**eGFR**	
	Coef (S.E.)	p	Coef (S.E.)	p	Coef (S.E.)	p
FGF19	-0.030 (0.219)	0.892	0.694 (0.154)	<0.0001	-6.54 (1.28)	<0.0001
β-Klotho	0.405 (0.137)	0.003	0.210 (0.123)	0.086	-2.90 (1.27)	0.023
Age	0.637 (0.173)	<0.0001	0.719 (0.130)	<0.0001	-10.95 (1.41)	<0.0001
Glucose	0.171 (0.153)	0.264	-0.467 (0.159)	0.003	3.31 (1.45)	0.023
Calcium	-0.004 (0.136)	0.974	-0.066 (0.114)	0.562	0.72 (1.26)	0.566
Potassium	0.071 (0.358)	0.844	0.132 (0.295)	0.655	-1.46 (1.27)	0.252
Sodium	0.632 (0.430)	0.142	-1.036 (0.342)	0.002	1.55 (1.27)	0.223
Hypertension	-0.181 (0.144)	0.208	-0.050 (0.114)	0.664	0.98 (1.32)	0.460
Diabetes	0.561 (0.181)	0.002	0.546 (0.154)	<0.0001	-5.82 (1.52)	0.0001
Dyslipidemia	0.250 (0.152)	0.099	0.696 (0.119)	<0.0001	-8.99 (1.36)	<0.0001
(Intercept)	-0.575 (0.184)	0.002	0.948 (0.130)	<0.0001	44.60 (1.26)	<0.0001

CKD, chronic kidney disease; Coef, coefficient; S.E, standard error; eGFR, estimated glomerular filtration rate.
